# Recombinant feline parvovirus infection of immunized tigers in central China

**DOI:** 10.1038/emi.2017.25

**Published:** 2017-06-07

**Authors:** Xingang Wang, Tongyi Li, Hongying Liu, Jimei Du, Feng Zhou, Yanming Dong, Xiuyuan He, Yongtao Li, Chuanqing Wang

**Affiliations:** 1 Department of Preventive Veterinary Medicine, College of Animal Husbandry and Veterinary Science, Henan Agricultural University, Zhengzhou 450002, Henan Province, China; 2 Zhengzhou Zoo, Zhengzhou 450008, Henan Province, China; 3 Department of Molecular Microbiology and Immunology, University of Missouri-Columbia, School of Medicine Bond Life Sciences Center, Columbia, MO 65211, USA

**Dear Editor,**

Feline panleukopenia is a contagious disease commonly characterized by severe hemorrhagic gastroenteritis and leukopenia, and it is associated with a high rate of morbidity in cats.^1^ The disease is caused by feline panleukopenia virus (FPLV), which belongs to the feline parvovirus subgroup within the genus *Parvovirus* (family *Parvoviridae*), together with canine parvovirus (CPV) and other parvoviruses of carnivores.^2^ FPLV can infect domestic cats and other Felidae species, as well as species of the families *Mustelidae, Procyonidae and Viverridae*.^3^ FPLV infections in large carnivores, such as captive tigers and lions, have been described based on clinical, serological and virological data.^4, 5, 6, 7^ However, the number of FPLV strains isolated from the adaptive carnivore species and the molecular information associated with these isolates are very limited. Clinical and etiological investigations are very important for discovering the mechanism(s) underlying the genetic evolution and pathogenicity of FPLV.

In this study, we report an outbreak of fatal FPLV infection among captive Southeast tigers (*Panthera tigris altaica*) in the Zhengzhou Zoo in central China. A novel FPLV strain was successfully isolated from these tigers. The tigers had been administered two doses of a vaccine (Nobivac Tricat, Intervet) against FPLV at three and four months of age. On 6 June 2016, a 5-month-old tiger presented with non-specific signs of infection, including fever, lethargy and anorexia. Subsequently, the tiger began to vomit and developed watery to hemorrhagic diarrhea later in the course of the disease, and transmitted the disease to nine additional tigers and lions (all between five to eight months of age) that were housed in the same or adjacent rooms. The animals failed to respond to antibiotic and supportive therapy, and five tigers died less than two days after the onset of diarrhea. The gross findings at necropsy in the tigers revealed diffuse fibrinous enteritis with dilatation of the intestine primarily affecting the ileum. The intestinal lumen contained a dense pinkish liquid with abundant epithelial debris and fibrin, as well as severe hemorrhagic enteritis with marked turgidity of the wall of the ileum. In addition, there was hemorrhaging and edema in the lungs, diffuse bleeding in the kidneys and bleeding spots and necrotic foci on the surface of the heart (Supplementary Figure S1).

To identify the major cause of death among the tigers, the presence of parvovirus, rotavirus, coronavirus and canine distemper virus in fecal, intestinal and blood samples of the dead tigers with enteritis was evaluated by PCR, RT-PCR, and sequencing and by inoculation of cell cultures for virus isolation. Our results showed that only FPLV was detected in the samples. The filtered homogenates of positive intestinal samples were used to inoculate a Crandell-Rees feline kidney cell monolayer as described previously.^6, 7^ Cytopathogenic effects were observed in infected Crandell-Rees feline kidney cells beginning 36 h post inoculation (Supplementary Figure S2). The presence of parvovirus in the supernatant of the cell cultures was confirmed by PCR. An FPLV strain, designated HN-ZZ1, was purified from the supernatant of the cell cultures by plaque assay, and the viral genome was sequenced by the Shanghai Sangon Biotechnology Company. The full-length genome of HN-ZZ1 comprises 4688 nucleotides (nt) and has been deposited in GenBank (accession number KX685354.1). Bioinformatic analysis of the viral sequence predicted that the HN-ZZ1 isolate encodes four proteins, including two nonstructural proteins, NS1 (nt 114–2120) and NS2 (nt 114–373 and 1846–2083), and two structural proteins, VP1 (nt 2127–2158 and 2223–4382) and VP2 (nt 2628–4382). The nucleotide sequence of HN-ZZ1 is closely related to viruses previously isolated from domestic cats in China and shows the highest identity (99.2%) with FPLV strain HRB-CS1, which was isolated from a dead domestic cat with enteritis in 2014 in Heilongjiang Province in northeastern China. Phylogenetic analysis of the whole genome of the HN-ZZ1 strain and 25 reference strains (Supplementary Table S1) showed that HN-ZZ1 was clustered in the FPLV branch but was also closely related to the CPV branch (Supplementary Figure S3).

Genetic recombination is generally considered to be a key mechanism underlying the generation and evolution of parvoviruses. Co-infection with multiple parvovirus strains has occurred, potentially facilitating recombination and leading to high genetic heterogeneity.^8^ Recently, a Japanese research group provided clear evidence of recombination between CPV and FPLV under field conditions.^9^ Interestingly, an analysis of the HN-ZZ1 sequence with the recombination detection programs RDP3 and GARD suggested that the HN-ZZ1 strain is a novel recombinant virus between CPV and FPLV. The RDP3 program identified nt position 2696 as an ending breakpoint, and a predicted recombination region was located around positions 1328 and 2696 of the alignment. Among the nucleotide sequence data for all 25 strains entered the RDP3 program, this part possessed the greatest similarity (99.3%) to the corresponding part of CPV-2b strain CPV-447 isolated from Germany (Supplementary Figure S4). The GARD program identified nt position 2558 as a putative breakpoint for recombination. The alignment was cut at this point, and separate phylogenies for each section of HN-ZZ1 were constructed. The first half of the fragment aligned with the clade containing CPV, while the latter half of the fragment aligned with the clade containing FPLV. In addition to the HN-ZZ1 strain isolated in the tigers, there is strong evidence supporting recombination events in the genomic sequences of MEV_LN-10 previously isolated from mink, and XJ-1 and HRB-CS1 previously isolated from cats in China.^10, 11, 12^

Phylogenetic analysis of the *VP2* gene of the HN-ZZ1 strain and 21 reference strains showed that the *VP2* gene of HN-ZZ1 aligned with the FPLV branch originating in Felidae and clustered in the FPLV branch. This study also indicated that the *VP2* gene of HN-ZZ1 showed a relatively distant relationship from those of the Purevax and Felocell vaccine strains. We speculated that the differences in the *VP2* genes between the wild type and vaccine strains may be one of the factors underlying the failure of vaccination against FPLV.^13^ Interestingly, the nucleotide sequence of the *NS1* gene was more closely related to the CPV branch originating in dogs and clustered with virus strains in the CPV branch (Figure 1A). We analyzed host-specific sites of key amino acids in the VP2 protein of the HN-ZZ1 strain and found that the amino acids at positions 80, 93, 103, 300, 305, 323, 564 and 568 of VP2 were identical to those of FPLV reference strains. However, the NS1 protein of the HN-ZZ1 strain had amino-acid characteristics of CPV, with the exception of the amino acid at position 248 (Figure 1B).^6^ An analysis using other evolutionary models, such as the Jukes-Cantor method, produced trees with nearly identical topologies (data not shown).

Our data herein suggest that HN-ZZ1 is a novel recombinant parvovirus between CPV and FPLV, and the recombinant virus might be epidemic in China. If this virus is epidemic, it will place wild and captive animals at increased risk. Although the role of this recombination event between CPV and FPLV in viral pathogenicity is unknown, our preliminary infection studies confirmed the high virulence of this recombinant virus. The intranasal infection studies in six 3-month-old cats with the HN-ZZ1 isolate resulted in anorexia, vomiting and fever in the affected animals at 2-day post infection. Subsequently, the cats began to display severe vomiting and developed watery to hemorrhagic diarrhea, which resulted in nearly 100% mortality in affected cats at 4–6 days post infection. Gross examinations showed that the affected cats were characterized by mild to severe fibrinous to fibrinomucoid enteritis with thickened mucosa, with bleeding spots on the surface of the heart, liver, lungs and spleen (data not shown). At present, the number of strains from Zoo animals that have been characterized by molecular methods are very limited, as can be observed by the scarce complete VP2 sequences available in GenBank. With the emergence and continuous increase of recombinant parvoviruses, our ongoing studies will investigate the geographic distribution of these viruses, monitor their recombination characteristics, identify their virulence determinants by reverse genetics and develop efficient strategies against these novel viruses.

## Figures and Tables

**Figure 1:**
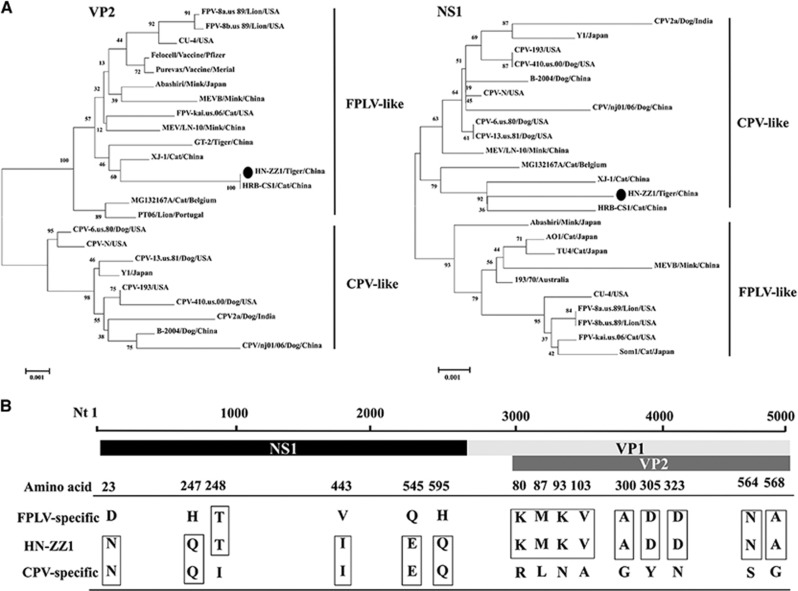
The results of the phylogenetic analysis of the *VP2* and *NS1* genes from the HN-ZZ1 strain and the deduced amino-acid sequence characterization. (**A**) Phylogenetic trees were inferred for the nucleotides of the *VP2* and *NS1* genes. The trees were generated by the neighbor-joining method with bootstrap tests of 1000 replicates using the MEGA 6.05 software. The HN-ZZ1 strain isolated in the current study is indicated (•). The GenBank accession numbers of the sequences are provided in Supplementary Table 1. (**B**) Amino-acid sequence characterization of the NS1 and VP2 proteins from the HN-ZZ1 strain.

## References

[bib1] Battilani M, Balboni A, Ustulin M et al. Genetic complexity and multiple infections with more Parvovirus species in naturally infected cats. Vet Res 2011; 42: 43. 21366901 10.1186/1297-9716-42-43 PMC3059301

[bib2] Truyen U. Evolution of canine parvovirus—a need for new vaccines? Vet Microbiol 2006; 117: 9–13. 16765539 10.1016/j.vetmic.2006.04.003

[bib3] Stuetzer B, Hartmann K. Feline parvovirus infection and associated diseases. Vet J 2014; 201: 150–155. 24923754 10.1016/j.tvjl.2014.05.027

[bib4] Steinel A, Parrish CR, Bloom ME et al. Parvovirus infections in wild carnivores. J Wildl Dis 2001; 37: 594–607. 11504234 10.7589/0090-3558-37.3.594

[bib5] Duarte MD, Barros SC, Henriques M et al. Fatal infection with feline panleukopenia virus in two captive wild carnivores (Panthera tigris and Panthera leo). J Zoo Wildl Med 2009; 40: 354–359. 19569486 10.1638/2008-0015.1

[bib6] Steinel A, Munson L, van Vuuren M et al. Genetic characterization of feline parvovirus sequences from various carnivores. J Gen Virol 2000; 81: 345–350. 10644832 10.1099/0022-1317-81-2-345

[bib7] Wang H, Jin H, Li Q et al. Isolation and sequence analysis of the complete NS1 and VP2 genes of canine parvovirus from domestic dogs in 2013 and 2014 in China. Arch Virol 2016; 161: 385–393. 26573526 10.1007/s00705-015-2620-y

[bib8] Battilani M, Balboni A, Giunti M et al. Co-infection with feline and canine parvovirus in a cat. Vet Ital 2013; 49: 127–129. 23564594

[bib9] Ohshima T, Mochizuki M. Evidence for recombination between feline panleukopenia virus and canine parvovirus type 2. J Vet Med Sci 2009; 71: 403–408. 19420841 10.1292/jvms.71.403

[bib10] Wang J, Cheng S, Yi L et al. Evidence for natural recombination between mink enteritis virus and canine parvovirus. Virol J 2012; 9: 252. 23110843 10.1186/1743-422X-9-252 PMC3495801

[bib11] Liu C, Liu Y, Liu D et al. Complete genome sequence of feline panleukopenia virus strain HRB-CS1, Isolated from a domestic cat in northeastern China. Genome Announc 2015; 3: e01556–14. 25814618 10.1128/genomeA.01556-14 PMC4384158

[bib12] Shackelton LA, Hoelzer K, Parrish CR et al. Comparative analysis reveals frequent recombination in the parvoviruses. J Gen Virol 2007; 88: 3294–3301. 18024898 10.1099/vir.0.83255-0 PMC3326350

[bib13] Truyen U, Parrish CR. Feline panleukopenia virus: its interesting evolution and current problems in immunoprophylaxis against a serious pathogen. Vet Microbiol 2013; 165: 29–32. 23561891 10.1016/j.vetmic.2013.02.005

