# Supplementary figures and images for: Recombinant feline parvovirus infection of immunized tigers in central China
Source: Emerg Microbes Infect. 2017 Jun 7;6(6):e42–. doi: 10.1038/emi.2017.25 (PMC5520303; doi:10.1038/emi.2017.25)

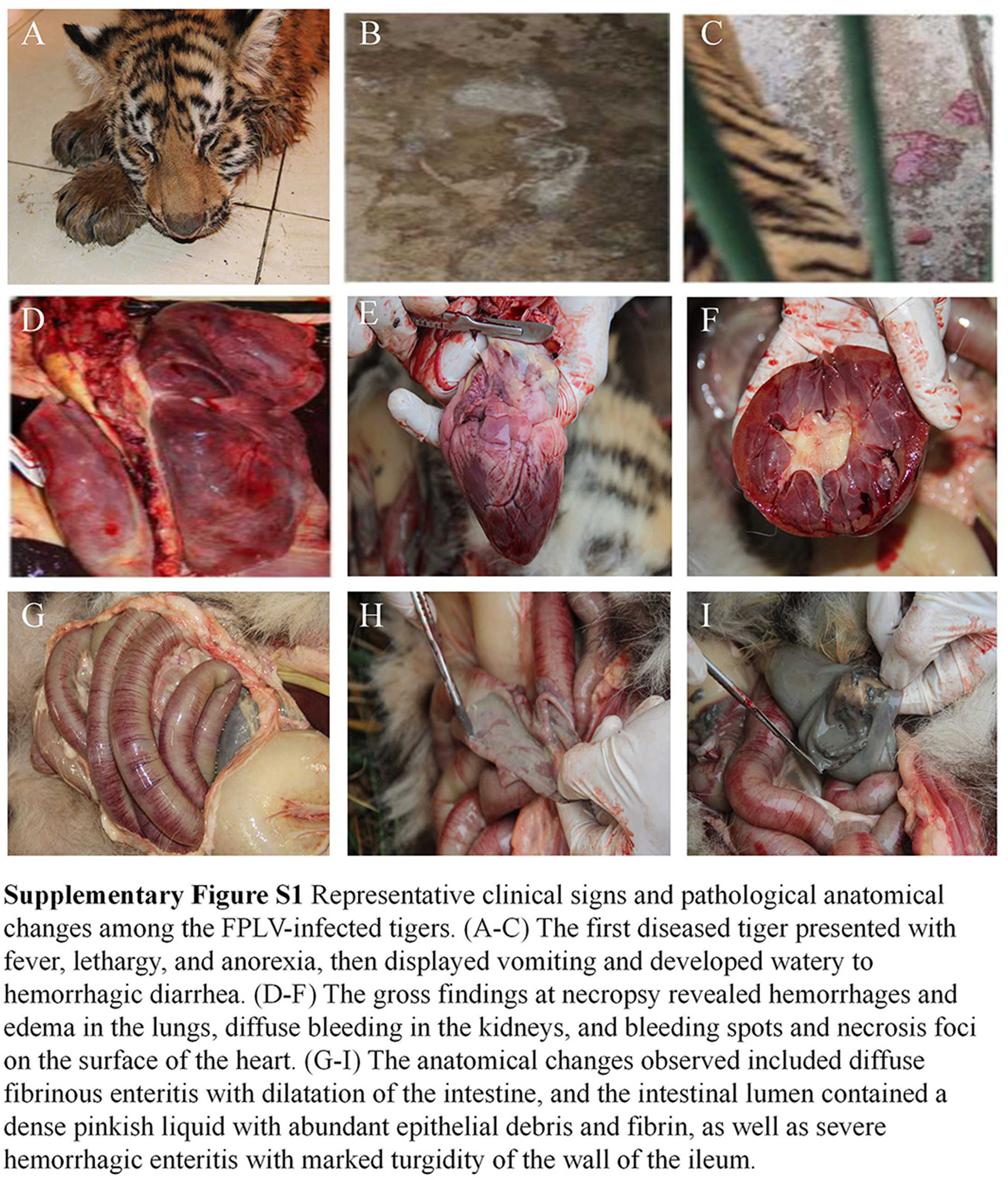

Supplement: Supplementary Figure S1 [file emi201725x1.tif]

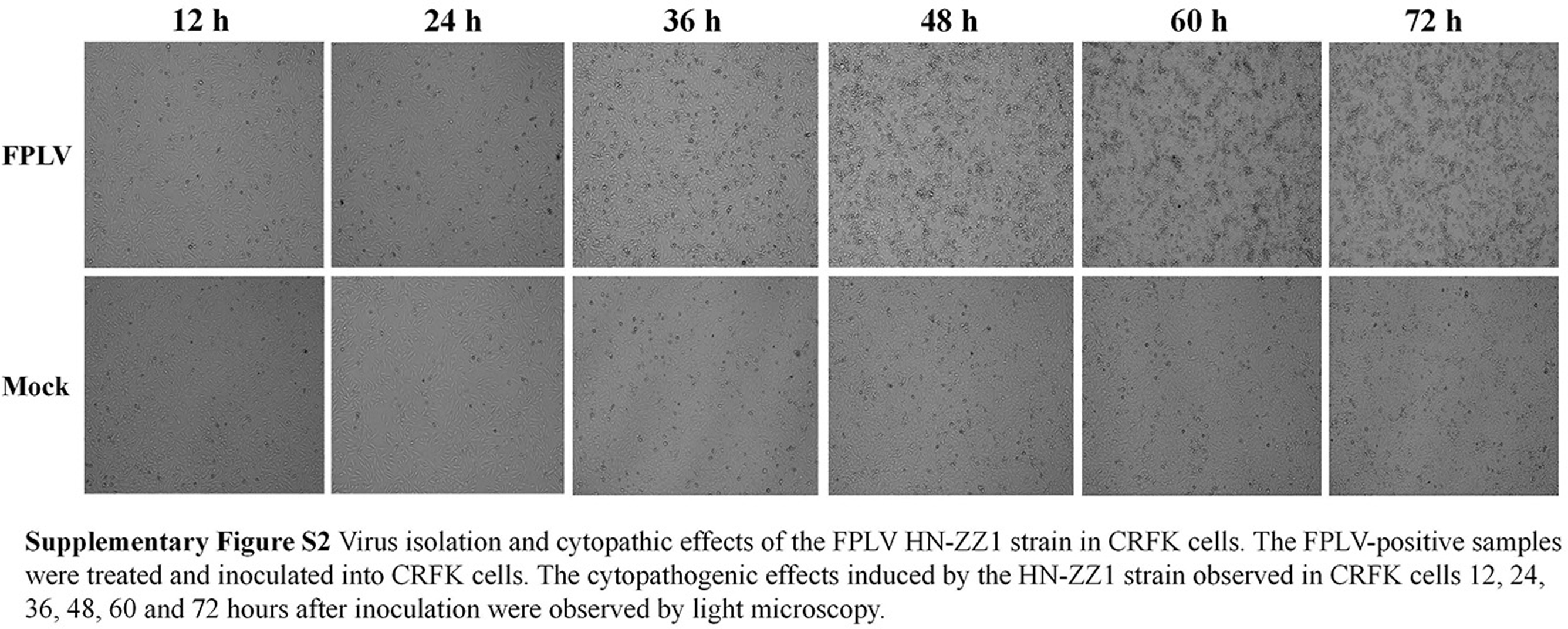

Supplement: Supplementary Figure S2 [file emi201725x2.tif]

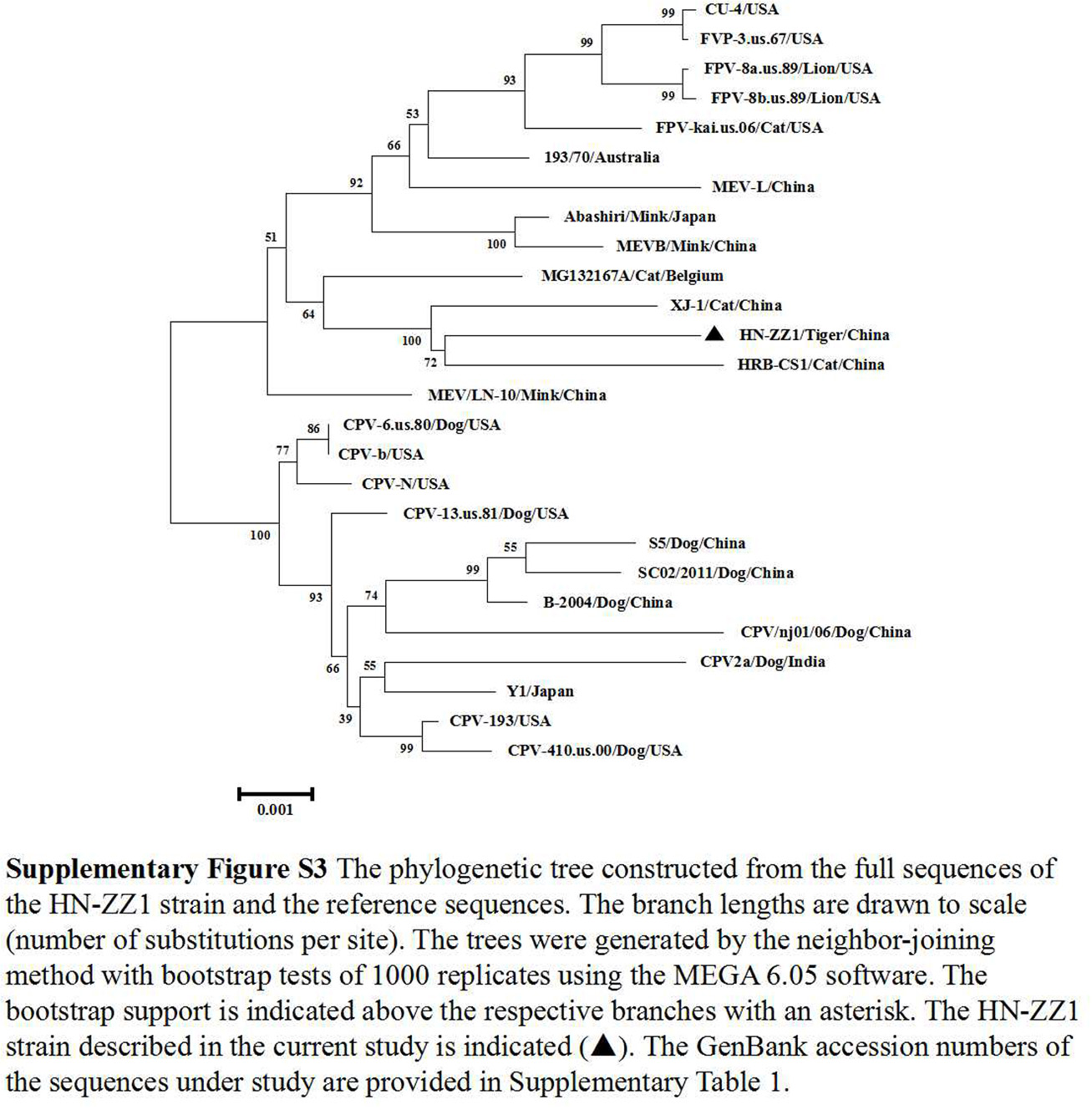

Supplement: Supplementary Figure S3 [file emi201725x3.tif]

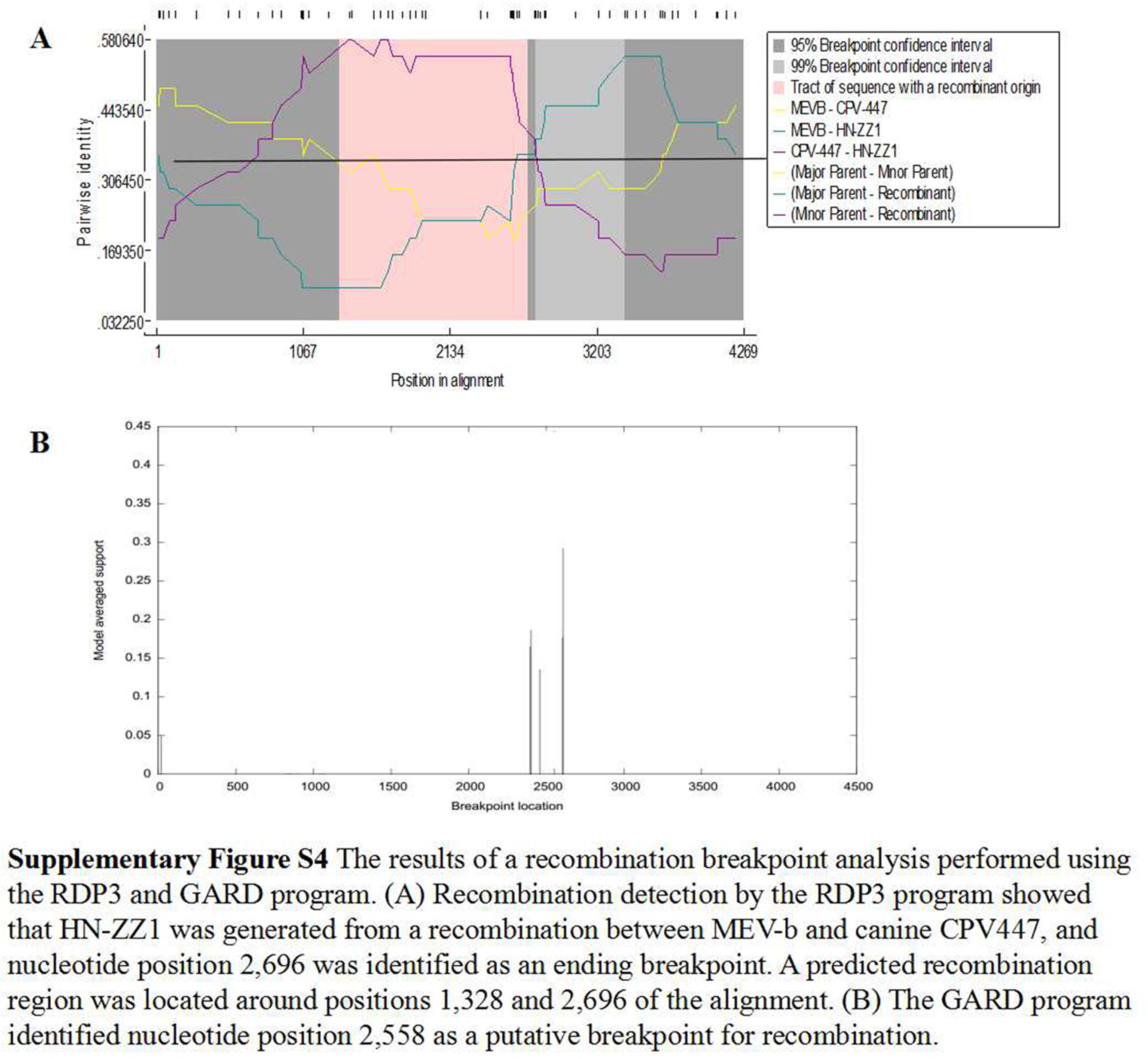

Supplement: Supplementary Figure S4 [file emi201725x4.tif]
