# Supplementary material for: Recombinant feline parvovirus infection of immunized tigers in central China
Source: Emerg Microbes Infect. 2017 Jun 7;6(6):e42–. doi: 10.1038/emi.2017.25 (PMC5520303; doi:10.1038/emi.2017.25)
Supplement: Supplementary Table S1 [file emi201725x5.doc]

**Supplementary Table S1** The nucleotide sequence accession numbers of the FPLV and CPV strains analyzed in this study.

| Strain | Accession number | Genetic type | Year submitted | Origin |
| --- | --- | --- | --- | --- |
| CPV-N | M19296 | CPV-2 | 1995 | USA |
| CPV447 | AY742934 | CPV-2 | 1995 | Germany |
| CU-4 | M38246 | FPLV | 1996 | USA |
| CPV-b | M38245 | CPV-2 | 1996 | USA |
| AO1 | AB000051 | FPLV | 1996 | Cat/Japan |
| TU4 | AB000067 | FPLV | 1996 | Cat/Japan |
| Som1 | AB000058 | FPLV | 1996 | Cat/Japan |
| Y1 | D26079 | CPV-2a | 2002 | Japan |
| B-2004 | EF011664 | new CPV-2a | 2004 | Dog/China |
| CPV2a | AJ564427 | new CPV-2a | 2004 | Dog/India |
| CPV-193 | AY742932 | new CPV-2b | 2005 | USA |
| CPV-193 | AY742932 | new CPV-2b | 2005 | USA |
| 193/70 | X55115 | FPLV | 2005 | Australia |
| GT-2 | AY955826 | FPLV | 2005 | Tiger/China |
| XJ-1 | EF988660 | FPLV | 2007 | Cat/China |
| Abashiri | D00765 | MEV | 2007 | Mink/Japan |
| CPV-13.us.81 | EU659118 | new CPV-2b | 2008 | Dog/USA |
| FPV-8a.us.89 | EU659113 | FPLV | 2008 | Lion/USA |
| FPV-8b.us.89 | EU659114 | FPLV | 2008 | Lion/USA |
| FPV-3.us.67 | EU659111 | FPLV | 2008 | USA |
| FPV-kai.us.06 | EU659115 | FPLV | 2008 | Cat/USA |
| CPV-410.us.00 | EU659119 | new CPV-2b | 2008 | Dog/USA |
| CPV-6.us.80 | EU659117 | CPV-2 | 2008 | Dog/USA |
| cpv/nj01/06 | EU310373 | CPV-2a | 2008 | Dog/China |
| Purevax | EU498680 | FPLV | 2008 | Vaccine/Merial |
| Felocell | EU498681 | FPLV | 2008 | Vaccine/Pfizer |
| MEVB | FJ592174 | MEV | 2009 | Mink/China |
| MEV/LN-10 | HQ694567 | MEV | 2011 | Mink/China |
| SC02/2011 | JX660690 | CPV | 2012 | Dog/China |
| S5 | KF638400 | CPV | 2013 | Dog/China |
| HRB-CS1 | KP280068 | FPLV | 2014 | Cat |
| MEV-L | KT899746 | MEV | 2015 | Mink/China |
| MG132167A | KP769859 | FPLV | 2015 | Cat/Belgium |
| PT06 | EF418568 | FPLV | 2015 | Lion/Portugal |
